# Progress in Otology

**Published:** 1900-06-23

**Authors:** 


### Progress in Otology.

(*Continued from page* 189.)

**Thrombosis of the Lateral Sinus.**—Thomas Barr and J. H. Nicoll[5] report an interesting case of this kind. The patient, aged 30, had had ear discharge for 15 years (left). The present illness began with violent earache, followed by frequent severe rigors, constant vomiting, and a temperature suggestive of pyæmia. The mastoid was opened and the tympanum cleared of pus, granulations, and cholesteatomata. The sinus appeared normal. Eleven days later Nicoll tied the jugular vein in the neck, not dividing it, and laid open and cleared out the purulent sinus in its whole extent. Ultimately the patient recovered. At the time of the last operation the patient had septic pneumonia with abscess and gangrene of the lung. Dr. Finlayson had examined the patient and found no signs in the chest indicative of a pulmonary cavity, though the quantity of pus expectorated pointed to this. On recovery the right side of the chest fell in, and there was diminution of expansion on movements of inspiration. To expose the sinus bone was removed from the mastoid to the torcular. Nicoll expects the gap to gradually fill up with bone. In criticising operations for this condition he pointed out that ligature of the jugular vein largely limited, though it did not remove entirely, the chances of general pyæmic infection. Without opening the sinus it was impossible to tell whether thrombosis existed. He had met with several cases where the sinus was free from granulation tissue externally, and exhibited pulsation and was collapsable by digital pressure, in which it had therefore been concluded that thrombosis had not occurred, and yet in these cases a post-mortem had shown that though there was a stream of fluid blood on the external side, on the deep side there had been adherent purulent thrombus. Therefore the sinus should be opened to decide the point. He recommended postponing the incision for 24 or 48 hours, unless the symptoms were too urgent, if a septic mastoid cavity had just been exposed and cleared of its contents, to avoid risk of infecting the sinus if found free from thrombus. In any case the bony cavities should be very thoroughly purified before opening the sinus. Moreover, it should not be opened unless the operator was prepared to proceed to the more or less extensive operation necessary for the complete removal of the purulent thrombus. Ligaturing the jugular vein without dividing it abolished a risk of infecting tissues near it, and the danger of the detachment of the ligatures through the struggling and restlessness of a delirious patient. A. R. Barker[6] records a case of sinus thrombosis, in which he opened the sinus and curretted upwards until blood flowed freely, and then tamponed with iodoform gauze. The lower portion was treated in the same manner, curetting well down into the jugular. Complete and uncomplicated recovery followed. This is specially interesting, because the jugular vein was not ligatured. Dundas Grant[7] records another case in which recovery followed although the jugular vein was not tied. The patient, a woman of 22, arrived at the hospital in an almost moribund condition, with a temperature of 103 deg., and had had repeated shiverings for a fortnight. On exposing the lateral sinus pus was found issuing from a hole in its wall. It was slit up freely, and a quantity of broken-down clot cleared out. On scraping finally, both in the backward and forward direction, normal-looking clot was reached, and the curetting was not proceeded with further. It is suggested that the normal clot acted like a ligature of the jugular. Arthur H. Cheatle[7] records yet another case of the same kind. He states that thrombosis in otitis may be due either to the passage of organisms from the middle ear through the wall of the sinus, the clot in that case readily breaking down and containing the bacteria which have given rise to it; or the blood may clot owing to "spread of the inflammation" from surrounding structures, when the coagulation is probably the effect of the transudation of toxins, and the clot is firm with little tendency to become disintegrated. In Cheatle's case, as in that mentioned above, the clot some distance above and below the point of infection was dark coloured and firm. The lower portion of the sinus was not made to bleed, that towards the torcular was. There had been one rigor before the operation, and there was one an hour after, but from that time progress was steady. Young[4] has reported another of these cases. Cheatle remarks that it seems that if a healthy-looking clot can be seen well below the breaking-down area, ligation of the vein is not necessary; but, in order that such a condition may be found, it must be well recognised that *one* rigor demands instant operation, the antrum being opened and the sinus examined. Cheatle attributes the giddiness of his patient to the disturbance of the intracranial circulation from the occlusion of the sinus. The boy had double optic neuritis, and though he was perfectly well, this neuritis was present three months after the operation.

**Otitic Meningitis.**—Bormans[6] gives an account of two cases of this affection, both treated by Quincke's puncture, in which recovery ensued. One patient was a boy, who represented the picture of true meningitis (with papillitis); the other, a woman, aged 45, with otitis and meningeal phenomena. In both Quincke's lumbar puncture was made twice, and in each the fluid removed contained staphylococci. The punctures every time produced relief.

**Otitic Abscess of the Brain.**—Ernest Waggett[8] has described a case of cerebellar abscess, treated successfully, and followed by sequestration of the cochlea. It was a case of chronic otorrhœa, with complete deafness. The man had vertigo and pains. A polyp was removed from the ear. Thirty-three days later it was necessary to open a cerebellar abscess. The operation performed was Dean's, because it was not certain whether there was a cerebellar or cerebral abscess. A cerebellar abscess was found in the region of the flocculus, as far forward as an abscess could be in the lateral lobe of the cerebellum; in fact, very near the apex of the petrous bone. He brought the case forward to show that there were two classes of cerebellar abscess. There was the abscess produced by infection through the lateral sinus, which was comparatively common; and there was the rarer affection where infection took place through the internal auditory meatus, affecting the anterior part of the lateral lobe of the cerebellum. He had seen two such cases, and read of others in the last two years. His patient rapidly recovered after the operation, and was walking about in three weeks. Owing to the man's critical condition at the time of operation, he had left dead bone which he had seen in the fundus, and it had gradually loosened in the ten months which had elapsed, and now had come away. Hausberg (Dortmand)[3] recently operated on two cases of cerebellar abscess, one of the cases, a child of 12, recovering. Hartmann also recently operated on a cerebellar abscess, the only symptoms of which were a diminution of outward movement of the eye, a little nystagmus, high fever, and violent headache. The patient died after temporary improvement.

[5] Glasgow Med. Jour., Jan., 1900. [6] Vide Laryngoscope, Jan., 1900. [7] Lancet, Jan., 1900. [8] Jour. Laryng., R., and O., Mar., 1900.

